# Management of bulbar conjunctival injury by honeybee sting: A case report of a retained honeybee stinger

**DOI:** 10.1016/j.ajoc.2022.101365

**Published:** 2022-01-26

**Authors:** Sarah Madison Duff- Lynes, Pamela Martin, Erich P. Horn

**Affiliations:** a University of Florida, Ophthalmology Department, 1600 SW Archer Rd, Gainesville, FL, 32608, USA; b Department of Ophthalmology, Malcolm Randall Department of Veterans Affairs Medical Center, Gainesville, FL, USA

## Abstract

**Purpose:**

To report a rare case of a bee sting to the conjunctiva of the eye in which the stinger remains in the subconjunctival space.

**Observations:**

We present the case of a fifty-five-year-old male who sustained a honeybee sting to the conjunctiva of his left eye after which some stinger remnants were left in place. He was initially treated with topical antibiotics, and topical and systemic steroids were added the next day. His visual acuity recovered fully with this regimen, despite later visualization of a retained bee stinger in the subconjunctival space.

**Conclusion and importance:**

Our experience suggests that though immediate removal of a stinger in the case of a bee sting to the eye is likely the safest approach, the long-term persistence of a bee stinger in the conjunctiva may not pose a threat to visual acuity and ocular health.

## 1 Introduction

Ocular injuries from bee stings are an uncommon occurrence in urban populations and optimal treatment has not yet been established. Bee stings to the eye may be extremely serious and threaten visual function. The range of complications are dependent on the ocular site of the sting and may include toxic keratopathy with severe, persistent corneal edema,^1^ keratouveitis, raised intraocular pressure, cataract formation, lens subluxation, hyphema, iris atrophy, optic neuritis, and toxic endophthalmitis.^2^ Frequently, the honeybee stinger is removed from the site of sting prior to medical presentation. However, on occasion, the stinger is present at the initial examination. Historically, due to concern for foreign body induced inflammation, the stinger has been typically removed from the ocular tissue immediately.^2^^,^^6^, ^7^, ^8^, ^9^, ^10^ We report a fifty-five-year-old Caucasian male who presented after being stung in the conjunctiva by a honeybee. Due to the initial report that the stinger was removed in total by the Emergency Department physician, immediate surgical exploration to identify and remove any remaining honeybee appendages was not performed. This is the first reported case of a bee sting to the conjunctiva with long-term retention of a honeybee stinger foreign body in the subconjunctival space.

## 2 Case report

A fifty-five-year-old male beekeeper presented to the Emergency Department (ED) after being stung in the left eye by a honeybee while mowing grass near his beehives several hours prior to his presentation. The ED physician reportedly removed the stinger from the left eye, then transferred the patient for further evaluation by the ophthalmologist on call.

The patient was experiencing severe left eye pain. Visual acuity (VA) was 20/20 without correction in the right eye (OD) and 20/25–2 in the left eye (OS). The intraocular pressures were normal at 10 and 9 mm of mercury (mmHg) in the right and left eyes, respectively, measured with a TonoPen tonometer. The pupils of both eyes responded appropriately to light without an afferent pupillary defect (APD). The right eye appeared normal. On examination of the left eye, an area of temporal conjunctival chemosis (less than one-by-one centimeter in size) was seen without subconjunctival hemorrhage. A fluorescein sodium sterile strip highlighted a small conjunctival defect. A small foreign body (that was presumed to be a stinger part) was seen subconjunctivally by manipulation of the conjunctiva after instillation of 1% tetracaine. Jeweler's forceps were used to remove the subconjunctival foreign body. The cornea did not appear to be injured. There was no anterior or posterior chamber inflammation or signs of infection. The vitreous was quiet and the retinal examination was normal without hemorrhages, retinal holes, or tears. The optic disc was unremarkable.

Due to the patient's significant pain with manipulation of the anesthetized conjunctiva and assurance from the ED physician that the stinger had previously been removed in the setting of good visual acuity, despite poor visualization with the chemotic conjunctiva, a more invasive surgical exploration of the conjunctiva was not conducted and it was decided to arrange for frequent follow-up appointments until the swelling improved. Moxifloxacin four times daily and erythromycin ointment nightly were started topically to prevent secondary bacterial infection. Tetanus prophylaxis was given.

The following morning, visual acuity remained relatively stable at 20/25 OS. The chemosis of the left eye had spread and an area of scattered subconjunctival hemorrhage was now present. A small foreign body was visualized at the slit lamp and, after applying 1% tetracaine, it was removed with forceps. The cornea remained clear and the anterior chamber was quiet. Due to the concern for inflammatory complications from a bee sting to the eye, the patient was started on prednisolone acetate 1% every 2 h while awake and 20 mg (mg) of oral prednisone daily. On this visit, another foreign body was briefly visualized with manipulation of the conjunctiva. It was discussed whether or not to remove this foreign body that, due to limited accessibility and visibility, would require conjunctival incision. Due to the improvement in the patient's pain and the stability of the visual acuity on examination, and the questionable existence of this possibly briefly-visualized foreign body, removal with conjunctival incision was not attempted.

**Fig. 1:**
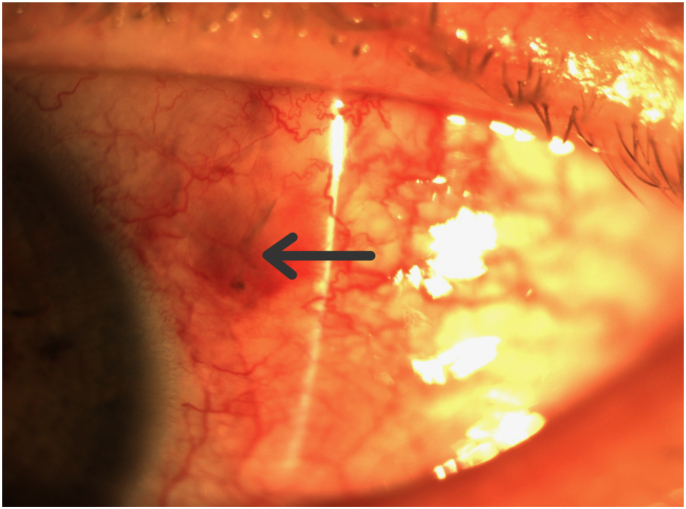
Palpebral conjunctiva of the left eye showing a subconjunctival foreign body, **[Arrow]** presumably a honeybee stinger, two weeks after the sting.

**Fig. 2:**
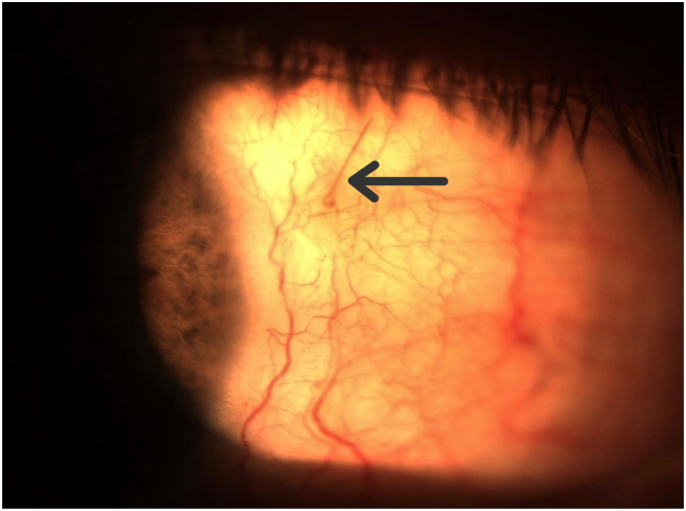
Palpebral conjunctiva of the left eye showing a subconjunctival foreign body, **[Arrow]** presumably a honeybee stinger, four and a half months after the sting.

At five and eight days after the initial trauma, the temporal chemosis continued to improve and the eye pain resolved. Moxifloxacin was discontinued and prednisolone acetate 1% was reduced to four times a day. As the temporal conjunctival chemosis resolved, the remaining foreign body was better visualized. The brownish-colored, slender and slightly curved foreign body was estimated to be about 2 mm (mm) long and appeared to be part of the honeybee stinger. A photograph of the left eye at two weeks shows what appears to be part of the stinger remaining in the subconjunctival space. **[**Fig. 1**].** The patient was offered a procedure to remove the remaining foreign body, but he refused due to resolution of all of his symptoms. The oral prednisone was reduced to ten mg daily, then discontinued after thirty days in total. Over this time, the patient's vision returned to 20/20 and all ocular topical medications were discontinued. At four-and-a-half months from the initial honeybee sting, the patient remained off of all ocular medications and was symptom-free with excellent vision. **[**Fig. 2**].** The patient continued to refuse surgical removal of the retained honeybee stinger from under the conjunctiva.

## 3 Discussion

**Fig. 3:**
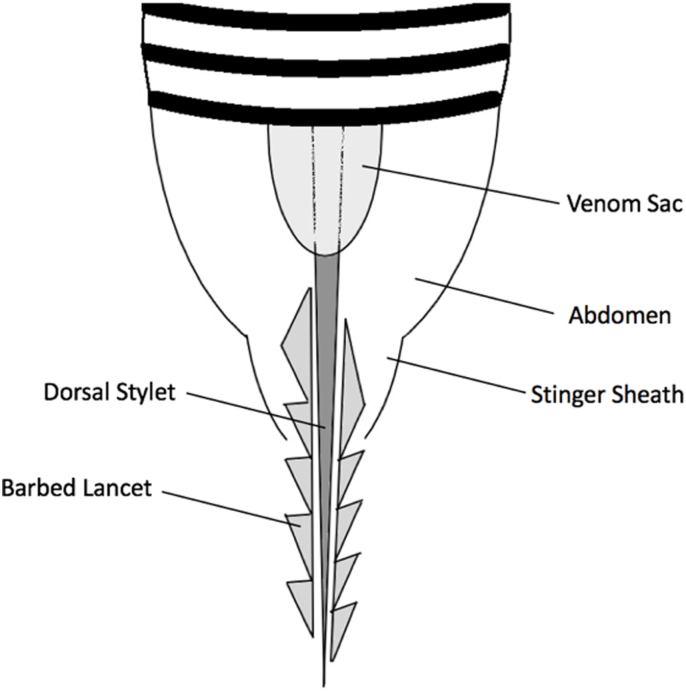
Anatomy of a honeybee stinger.

Bee stings to the eye, while uncommon, can have devastating consequences to both the patient and the bee. When the honeybee stings, its barbed stinger is embedded in the victim. Once the barbed stinger is embedded, it cannot be retracted. Instead, part of the attached abdomen is torn away, which kills the bee. The stinger remains embedded in the victim and injects venom released from the bee's venom sac, which continues to contract following its separation from the bee's abdomen. An estimated 90% of the venom is injected in the first 20 s and, if the stinger is not removed within a minute, all of the venom has been delivered.^3^ The combination of the trauma from the stinger and the inflammation from the venom damages ocular tissues and causes immediate pain. The honeybee stinger consists of three parts, a dorsal stylet and two barbed lancets on either side, which together form a chitinous tube through which venom passes into the wound. Complete removal of the stinger necessitates removal of all three parts, made more difficult by the lancets' multiple backward-facing barbs and small diameter. **[**Fig. 3**].** In this case, the referring ED physician removed a foreign body from the patient's left eye. Subsequently, two additional foreign bodies were removed from the subconjunctival space by ophthalmologists using slit lamp magnification. Finally, after chemosis and subconjunctival hemorrhage had resolved, an additional bee sting-related foreign body was clearly visible (see Fig. 1). While possible that the beekeeper was stung by multiple bees, it is far more likely that the ED physician removed the honeybee's abdominal appendages, including the venom sac, from the conjunctiva and the ophthalmologists each removed one of the two barbed lancets, which comprise the stinger along with the single long stylus which remains in the patient's subconjunctiva. When removing a bee stinger from a patient's eye, an understanding of the peculiar anatomy of the honeybee worker may guide the practitioner to look for and remove all parts of the stinger.

The honeybee stinger complex is an efficient, pointed delivery system for potent toxins, which are likely responsible for the chemosis and intense ocular pain experienced by the patient in this case. Honeybee venom consists of both alkaline and acidic components, including non-enzymatic polypeptides, enzymes, and biological amines. About half of the venom is melittin, which causes cell membrane disruption and denaturing of proteins, likely responsible for cataract formation, zonulysis, and lens dislocation.^4^ Apamin, a minor component of venom, is a neurotoxin that may disrupt potassium ion channels and may cause focal optic nerve demyelination in toxic optic neuritis. Phospholipase A2 and B are hydrolytic enzymes in bee venom which cause cytolysis and hemolysis.^1^

Most published cases in the literature have reported bee stings to the cornea.^2^ This could be due to publication bias on account of the severe ocular inflammation and tissue trauma that these corneal stings can cause. Alternatively, they may be more numerous than conjunctival stings, since the majority of the conjunctiva is covered (and largely protected) by the lids even when the eye is open. Using ImageJ, a graphic analysis interface, and a reference photograph of the human eye, 60% of the surface area of the eye vulnerable to bee sting is comprised of the cornea; the remaining 40% is conjunctiva. Corneal complications include toxic keratopathy with severe, sometimes permanent, corneal edema^1^ and keratouveitis^2^ which may require corneal transplantation to restore useful vision.

A Pubmed search was performed on December 13th, 2020 using the search results “bee” “sting” and “eye.” Of the thirty-seven previous publications of ocular bee stings, which included fifty-eight reported cases,^2^^,^^5^ only six publications of bee stings to the conjunctiva have been reported.^2^^,^^6^, ^7^, ^8^, ^9^, ^10^

In four of the six publications, the bee stinger was not seen at the initial examination.^6^, ^7^, ^8^^,^^10^ Each case varied in its presentation and course. Isawumi and Hassan^6^ reported a bee sting to the conjunctiva and sclera resulting in purulent eye discharge, chemosis, a ciliary staphyloma, and mild scleritis. A topical antibiotic and non-steroidal anti-inflammatory drug (NSAID), and an oral NSAID and steroid were used for successful treatment. Choi and Cho^10^ reported a bee sting to the conjunctiva that was originally treated with topical steroids prior to deterioration of VA to light-perception (LP) only. Corneal edema, conjunctival injection, inflammation of the anterior chamber, in addition to an afferent pupillary defect and optic disc swelling, were reported. Intravenous (IV) and oral steroids, a topical cycloplegic, a topical antibiotic, and a topical steroid were used for treatment, and the visual acuity ultimately returned to normal. Rishi and Rishi^8^ reported a case of anterior uveitis, vitritis, optic disc swelling, and cilio-choroidal detachment following a conjunctival bee sting which resulted in 20/20 visual acuity with chorioretinal atrophy in the area of prior choroidal detachment.

In only two of these six publications were the bee stingers seen at initial examination.^2^^,^^9^ In both of these cases, the stingers were immediately removed. No retained stingers were found on follow-up examination. Lin et al.^9^ reported a case of a bee sting to the conjunctiva near the limbus with a stinger remaining on initial examination. The stinger was removed immediately. Chemosis and conjunctival hyperemia were present, but no corneal or anterior chamber reaction occurred. Semler-Collery et al.^2^ also reported a case of a bee sting with retention of the stinger at the conjunctival site. There was conjunctival hyperemia and a conjunctival defect without apparent corneal or scleral injury, intraocular inflammation, or infection. The stinger was immediately removed in this case as well. A topical antibiotic and topical steroid were used without further complications.

In the medical literature, there are no cases reporting long-term retention of a honeybee stinger in the subconjunctival space, as in our case. Had the foreign body been visualized on initial examination, it would have been prudent to remove the presumed honeybee stinger, as foreign bodies may cause inflammation. Other articles state that stingers may continue to contain or release venom.^9^ While the honeybee apparatus continues to secrete venom after separation from the bee, it appears that all venom is released within the first minute after the sting.^3^ Thus, if a patient presents with a stinger in the eye or surrounding tissue and several minutes have passed, it is unlikely that any venom will continue to be secreted from the retained stinger. However, if the foreign body is easily accessible, it would be prudent to remove it since its presence may cause inflammation or infection. In our case, steroids were prescribed and a tetanus shot was given. Of the two previous cases of conjunctival stingers present on initial examination prior to removal^2^^,^^9^ neither case was treated with oral steroids, and only one of the two cases was treated with topical steroids, both with excellent visual outcomes. Choi and Cho's case of a conjunctiva sting without an initially retained stinger^10^ waited until vision decreased to start oral steroids and also ended with an excellent visual outcome. Perhaps in our case, we could have avoided topical or oral steroids due to the lack of intraocular inflammation on initial examination, but chemosis and continued pain prompted more aggressive anti-inflammatory treatment. In regards to a tetanus vaccine, there are no reported cases of tetanus infection occurring after a bee sting. For the purposes of tetanus prevention, honeybee stings can be considered “clean.” However, in this case a tetanus shot was administered.

Despite no other reported cases of long-term retention of bee stingers in the conjunctiva, there are reports of bee stinger retention in other parts of the eye without complications. Rai et al.^11^ reported a retained bee stinger in the peripheral cornea to sixteen-months follow-up without complications. Gilboa at el.^12^ reported a retained wasp stinger in the anterior chamber and anterior lens capsule stable after twenty-eight years of follow-up. Strobel^13^ reported a retained bee stinger in the posterior cornea and anterior chamber without complication to twenty-one years of follow-up. Finally, Sá et al.^14^ reported a case of a bee stinger embedded in a lens for five years without intraocular inflammation and only mild cataract. These cases highlight that once venom is neutralized, it may be possible for a bee stinger to be retained safely in ocular tissue.

While a patient may not be able to distinguish whether they were stung by a bee or a wasp (and even published articles may conflate the two), a bee sting may have a better outcome, presumably due to different toxins, but this should be further investigated.^12^^,^^15^ Due to this patient being a beekeeper and being stung while mowing grass near his beehives, this stinger was presumed to be from a honeybee.

## 4 Conclusion

Optimal management of a bee sting to the eye has yet to be fully elucidated, despite a growing collection of case reports in the literature. We report the first case of a bee sting to the conjunctiva with retention of a foreign body, a honeybee stinger, in the subconjunctiva to four-and-a-half months of follow-up without complications. Despite our patient's lack of complications, we would encourage careful clinical consideration of removing all stinger parts if it can be done without an invasive operation. If not, there is now good evidence of long-term tolerability of retained bee stingers in the conjunctiva and cornea. Although some cases similar to ours have had good outcomes without topical or systemic steroids, if any signs of intraocular inflammation are seen on examination or if there is any decrease in vision, topical steroid therapy should be initiated immediately and systemic steroid therapy considered unless there is infection or other contraindication. Prior reports have described bacterial keratitis following bee stings^11^ and presented culture data from insect stings that 14% are contaminated by bacteria.^16^ Use of broad-spectrum topical antibiotics as soon as possible after a bee sting would seem prudent as prophylaxis against secondary infection. Tetanus vaccination is not necessary after a bee sting if a patient is up to date with his or her vaccination.

## Funding

Funding was provided in part by an unrestricted grant from 10.13039/100001818Research to Prevent Blindness.

## Declaration of interests

☒ The authors declare that they have no known competing financial interests or personal relationships that could have appeared to influence the work reported in this paper.

☐The authors declare the following financial interests/personal relationships which may be considered as potential competing interests:

## Declaration of competing interest

There are no conflicts of interest to disclose.
